# TIPE2 mRNA overexpression correlates with TNM staging in renal cell carcinoma tissues

**DOI:** 10.3892/ol.2013.1388

**Published:** 2013-06-10

**Authors:** ZONGLIANG ZHANG, HAIYAN QI, SICHUAN HOU, XUNBO JIN

**Affiliations:** 1 School of Medicine, Shandong University, Jinan, Shandong 250012;; 2 Department of Urology, Qingdao Municipal Hospital, Qingdao, Shandong 266071;; 3 Minimally Invasive Urology Center, Provincial Hospital Affiliated to Shandong University, Shandong University, Jinan, Shandong 250021, P.R. China

**Keywords:** tumor necrosis factor α-induced protein-8 like-2, type I interferon, myxoma resistance protein 1, renal cell carcinoma, TNM staging

## Abstract

Tumor necrosis factor α-induced protein-8 like-2 (TIPE2) is a gene that maintains immune homeostasis. The aim of the present study was to determine whether TIPE2 is associated with renal cell carcinoma (RCC) progression. The mRNA expression levels of TIPE2 and myxoma resistance protein 1 (MX1; a type I interferon-inducible gene) were investigated in renal cancer tissues. A total of 46 patients who fulfilled the RCC criteria of the World Health Organization (WHO; revised in 2004) and 39 control patients with renal contusions requiring surgical extraction were enrolled. Tumor and normal renal tissues were sampled from all subjects through surgery. Total RNA was extracted and the expression of the TIPE2 and MX1 genes was evaluated by quantitative polymerase chain reaction (qPCR) analysis. TIPE2 mRNA expression was significantly upregulated in the RCC patients compared with the controls (P=0.001), while MX1 mRNA expression was decreased in the RCC patients compared with the controls (P=0.0001). Furthermore, the TIPE2 mRNA expression levels were positively correlated with TNM staging (r=0.803, P=0.001) and showed a negative correlation with the MX1 mRNA expression levels (r=−0.763, P=0.001) in the RCC patients. These results indicate that the increased expression of the TIPE2 gene is markedly associated with RCC and suggests an important role for the TIPE2 gene in the pathogenesis of RCC.

## Introduction

Renal cell carcinoma (RCC) is the most common malignant tumor of the kidney and its incidence has been increasing worldwide (1). Despite extensive clinical trials, the pathogenesis and determination of prognoses for patients with RCC have remained poor. Although the pathogenesis of RCC is not completely understood, the dysfunction of immune regulation mechanisms, such as abnormalities of the immune homeostasis-maintaining genes, appears to be an important contributor to the development of RCC (2,3).

Two classes of immune homeostasis-maintaining genes have been identified (4). The first class have functions in limiting the strength of immune cell activation and expansion. The second class controls cell death and includes Fas, Bim, Bax and caspases 8 and 10 (5). The majority of genes that maintain immune homeostasis by controlling cell death are also involved in the pathogenesis of RCC. Tumor necrosis factor-α-induced protein (TNFAIP) 8 like-2 (TNFAIP8L2 or TIPE2), a negative regulator of innate immunity and cellular immunity, shares considerable sequence homology with members of the TNFAIP8 family (6). It is preferentially expressed in lymphoid- and marrow-derived cells and its deletion in mice leads to multi-organ inflammation. TNFAIP8 is also aberrantly expressed in various human cancer cell lines, with higher levels in K562 chronic myelogenous leukemia cells, MOLT 4 lymphoblastic leukemia cells and A549 lung carcinoma and lower levels in SW480 colorectal adenocarcinoma cells (7). In addition, TNFAIP8 expression is induced by TNF-α, high glucose stimulation and activation of nuclear factor (NF)-κB in various cells (7–11). At present, little is known about the mechanism behind the role of TNFAIP8 in carcinogenesis. The common hypothesis is that TNFAIP8 may be an inhibitor of caspase-mediated apoptosis. The overexpression of TNFAIP8 in cancer cells may inhibit TNF-induced apoptosis by inhibiting caspase-8 and caspase-3 activity (7,9), while the depletion of TNFAIP8 enhances cell death (10,12). Considering that normal expression levels of the TIPE2 gene in the immune system are essential for maintaining immune homeostasis, we hypothesized that the expression of the TIPE2 gene in RCC patients may be different from that of normal individuals and thus may be involved in the pathogenesis of RCC. Therefore, in the present study, the TIPE2 mRNA expression levels in cancer tissue samples from RCC patients were compared with controls, and the correlations between TIPE2 mRNA expression levels and the TNM staging of RCC and the mRNA expression levels of myxoma resistance protein (MX1), an interferon (IFN)-I-inducible gene, were analyzed (13). The upregulation of TIPE2 mRNA expression was observed in the RCC patients and was markedly associated with the IFN-I levels and TNM staging of RCC. These findings suggest that TIPE2 may be involved in the pathogenesis of RCC.

## Materials and methods

### Human subjects

A total of 46 patients with clear cell RCC fulfilling the RCC criteria of the World Health Organization (WHO; revised in 2004) (14) were enrolled in the present study. As controls, 39 gender- and age-matched renal contusion patients who required surgical extractions were recruited. None of the control patients had any tumors or other abnormal conditions. The RCC staging was assessed with the TNM staging system for kidney cancer revised by the American Joint Committee On Cancer (AJCC) (15). Tumor and control renal tissues were sampled from all patients by surgery. All 46 patients were confirmed to have RCC through pathological examinations. This study was approved by the ethics committee of Qingdao Municipal Hospital, Qingdao, China. All patients gave informed written consent prior to the initiation of the study. The characteristics of the patients are shown in Table I.

### Laboratory assessments

For the RCC patients, the biochemical parameters of the serum, including lactate dehydrogenase (LDH), calcium (Ca), blood urea nitrogen (BUN) and creatinine (Cr), were analyzed using an automatic biochemical analyzer (Hitachi P7600; Hitachi, Tokyo, Japan). The hematological indices of the blood, including levels of red blood cells (RBC), white blood cells (WBC), hemoglobin (HGB) and platelets (PLT), were analyzed by an automatic hematological analyzer (Sysmex XS-800i; Sysmex, Kobe, Japan). The coagulation parameters of prothrombin time (PT) and activated partial thromboplastin time (APTT) were analyzed by an automatic coagulation analyzer (Sysmex CA7000, Sysmex, Kobe, Japan). Erythrocyte sedimentation rates (ESR) were analyzed with a MONITOR-J+ analyzer (Electa Lab Srl, Forli, Italy).

### RNA and cDNA preparation from RCC tissues and controls

Tumor and control renal tissues were sampled from all the subjects through surgery. The samples were pulverized using liquid nitrogen. Total RNA was extracted from the samples using TRIzol (Invitrogen, Carlsbad, CA, USA) and treated with RNase-free DNase (Sangon Biotech, Shanghai, China) to remove genomic DNA contamination. The RNA (1 *μ*g) was reverse transcribed to cDNA using a reverse transcription system kit (Sangon Biotech).

### Quantitative polymerase chain reaction (qPCR) analysis

The expression of the TIPE2 gene was evaluated by qPCR. The serum level of IFN-1 was quantified by qPCR measurements of the mRNA expression of the IFN-I inducible gene MX1. Primers were synthetized as described by Li *et al* (4). The primers for TIPE2 were as follows: forward, 5′-GGAACATCCAAGGCAAGACTG-3′ and reverse, 5′-AGCACCTCACTGCTTGTCTCATC-3′. The level of β-actin mRNA was also detected as an internal control for each sample. The primers for MX1 were as follows: forward, 5′-TGCTTATCCGTTAGCCGTGG-3′ and reverse, 5′-CGCCAGCTCATGTGCATCT-3′. For β-actin, the following primers were used: forward 5′-GACTACCTCATGAAGATCCTCACC-3′ and reverse, 5′-TCTCCTTAATGTCACGCACGATT-3′. qPCR was performed using the SYBR Green I Real-Time PCR kit in accordance with the instructions of the manufacturer (Takara, Dalian, China) in an ABI PRISM 7500 Sequence Detection System (Perkin-Elmer, Norwalk, CT, USA). The amplification conditions were as follows: 95°C for 10 sec, followed by 40 cycles of 95°C for 5 sec and 60°C for 41 sec. Each sample was run in triplicate. The PCR products were run on an agarose gel and all cases were confined to a single band of the expected size. A melting curve analysis was also performed to ensure the specificity of the products. The relative mRNA expression levels of TIPE2 and MX1 were determined using the comparative (2^−ΔΔCt^) method.

### Statistical analysis

Statistical analysis was performed using the SPSS 13.0 software (SPSS, Inc., Chicago, IL, USA). The data are expressed as the mean ± standard deviation. The differences in TIPE2 or MX1 mRNA levels between the subject groups were analyzed independently using Student’s t-test. A correlation analysis was performed using Spearman’s rank test. P<0.05 was considered to indicate a statistically significant difference. All figures were created with GraphPad Prism software, version 5.0 (GraphPad Software, Inc., La Jolla, CA, USA).

## Results

### General condition of patients

The clinical manifestations, demographic characteristics, TNM staging and laboratory measurements are shown in Table I.

The median hematological indices and biochemical parameters, including LDH, Ca, BUN and Cr levels of the RCC patients and controls all remained similar. No significant differences in general condition were observed between the RCC patients and controls.

### Quantification of TIPE2 mRNA expression in RCC tissues and controls by qPCR

**Figure 1.**
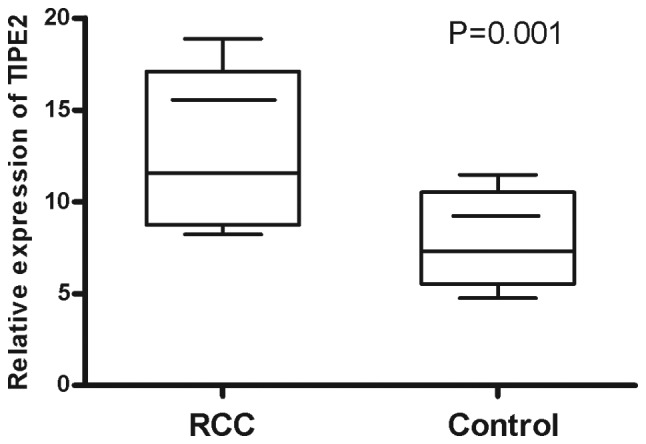
TIPE2 mRNA expression levels in RCC tissues and controls. Quantitative polymerase chain reaction (qPCR) was performed to quantify the expression of the TIPE2 gene from the RCC patients (n=46) and healthy controls (n=39). Box plots show the 25th and 75th percentiles. Horizontal lines within the boxes show the medians and short horizontal lines indicate the means (11.58 in the patient group and 7.32 in the control group). Vertical bars indicate the 10th and 90th percentiles. There was a significant increase in the TIPE2 expression of the RCC patients compared with the healthy controls (P=0.001). TIPE2, tumor necrosis factor-α-induced protein 8 like-2; RCC, renal cell carcinoma.

**Figure 2.**
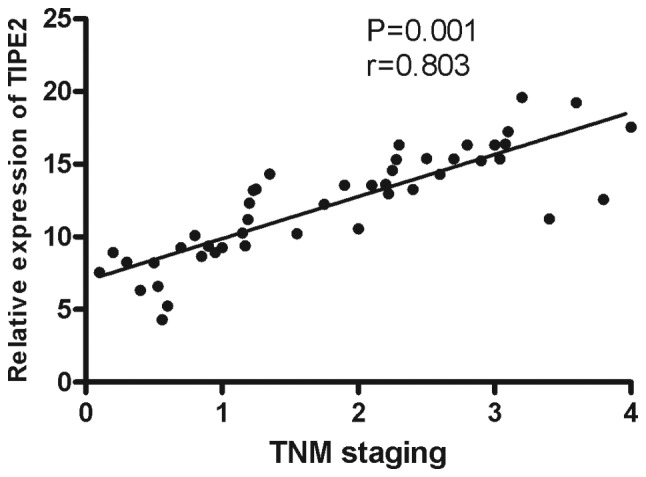
Positive correlation between TIPE2 mRNA expression levels and TNM staging among all renal cell carcinoma (RCC) patients (n=46). TIPE2, tumor necrosis factor-α-induced protein 8 like-2.

The expression levels of TIPE2 mRNA in the renal tumor tissue from 46 RCC patients and 39 gender- and age-matched controls were examined using qPCR. The results showed that the relative expression levels of TIPE2 mRNA (11.58±3.75) in the RCC patients were significantly higher compared with the controls (7.32±3.93; P=0.001; Fig. 1). The correlations between TIPE2 expression and demographic characteristics, clinical manifestations and laboratory parameters were also analyzed. The results demonstrated that the TIPE2 mRNA expression levels were positively correlated with TNM staging (r=0.803, P=0.001; Fig. 2). No statistically significant associations were observed between the TIPE2 mRNA expression levels and other characteristics, clinical manifestations or laboratory parameters among the RCC patients.

### Quantification of MX1 mRNA expression in RCC tissues and controls by qPCR

**Figure 3.**
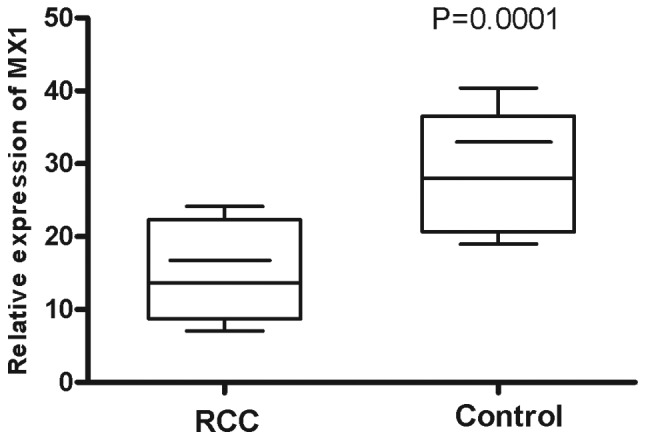
MX1 mRNA expression levels in RCC tissues and controls. Quantitative polymerase chain reaction (qPCR) was performed to quantify the expression of the MX1 gene from the RCC patients (n=46) and healthy controls (n=39). Box plots show the 25th and 75th percentiles. Horizontal lines within the boxes show the medians and short horizontal lines indicate the means (13.65 in the patient group and 27.96 in the control group). Vertical bars indicate the 10th and 90th percentiles. There was a significant increase in MX1 expression in the RCC patients compared with the healthy controls (P=0.0001). MX1, myxoma resistance protein 1; RCC, renal cell carcinoma.

**Figure 4.**
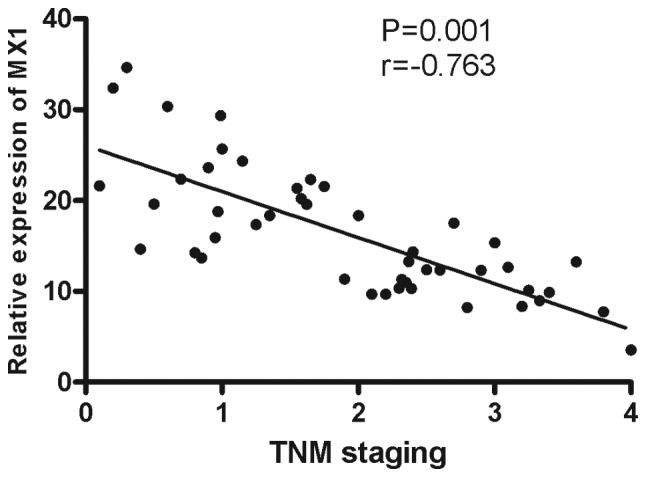
Negative correlation between MX1 mRNA expression levels and TNM staging among all renal cell carcinoma (RCC) patients (n=46). MX1, myxoma resistance protein 1.

Since all members of the IFN-I family bind to the same receptor complex, measuring the expression of IFN-I-inducible genes, such as MX1, by qPCR permits total IFN-I production (all isoforms) to be estimated (2). MX1 mRNA expression levels were examined in the renal tumor tissue samples from 46 RCC patients and 39 controls using qPCR. It was observed that the mean relative MX1 mRNA expression level (13.65±7.34) of the RCC patients was lower compared with the controls (27.96±9.98; P=0.0001; Fig. 3) and a negative correlation was observed between MX1 mRNA expression and TNM staging (r=−0.731, P=0.001; Fig. 4).

### Correlation between TIPE2 mRNA expression and MX1 mRNA expression in RCC tissues

**Figure 5.**
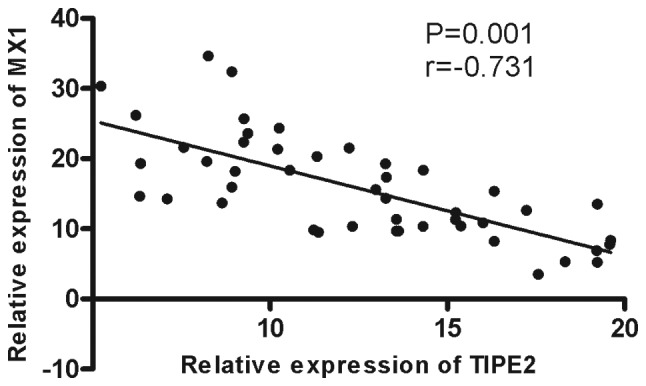
Negative correlation between the TIPE2 and MX1 mRNA expression levels among all renal cell carcinoma patients (n=46). TIPE2, tumor necrosis factor-α-induced protein 8 like-2; MX1, myxoma resistance protein 1.

Since IFN-I is important in the pathogenesis of RCC and the MX1 mRNA level represents the level of IFN-I, the association between TIPE2 and MX1 expression was investigated. The results showed a negative correlation between TIPE2 and MX1 mRNA expression (r=−0.763, P=0.001; Fig. 5). This indicates that TIPE2 mRNA expression levels are negatively correlated with the levels of IFN-I in the renal tumor tissues of RCC patients.

## Discussion

Immune homeostasis is maintained by multiple immune regulation genes that are unable to compensate for each other (4). The breakdown of immune homeostasis caused by the dysfunction of these genes contributes to tumor pathogenesis. TIPE2 is a negative regulator of innate and cellular immunity (5). In the present study, the upregulation of TIPE2 expression was demonstrated via qPCR and western blotting in RCC patients, and a positive correlation between TIPE2 expression and the TNM staging of RCC was reported for the first time.

Studies have indicated that TNFAIP8 is an apoptosis regulator which may be involved in oncogenesis (7,12,16–18). Upregulation of TIPE2 expression in RCC patients may cause an abnormal resistance to apoptosis, which would ultimately lead to cancer. Similar to several other regulators of apoptosis, TNFAIP8 contains a death effector domain (DED) and is able to inhibit caspase-mediated apoptosis (7). The majority of studies support the hypothesis that TNFAIP8 is able to inhibit TNF-α-induced caspase activation and apoptosis (7,10,12). Experimental evidence also supports the theory that TNFAIP8 is an oncogene in human cancers. TNFAIP8 overexpression has frequently been observed in several types of cancer tissues, suggesting that TNFAIP8 may be significant in oncogenesis (12,16,19). The evaluation of a limited number of clinical specimens has revealed higher expression levels of TNFAIP8 protein in human breast cancer and RCC tissues compared with matched normal adjacent tissues (16). The direct role of TIPE2 in apoptosis remains unclear. TIPE2 was considered to contain a DED or DED-like domain, but its structure and topology differ from that of the DED domains contained in caspase-8 or c-FLIP (20). It has been shown that TIPE2 binds to the Ras-interacting domain of the RalGDS family of proteins, which are essential effectors of activated Ras (22). RT-PCR revealed no significant differences in TIPE2 mRNA between hepatocellular carcinoma (HCC) and adjacent tissues (21). The development of human HCC is associated with the downregulation of TIPE2 (21). This result is possibly associated with inflammation since chronic HBV infection is a major cause of HCC. The direct correlation between increased TIPE2 expression and tumorigenesis in RCC patients requires further investigation.

The overexpression of the TIPE2 gene may cause the depression of proinflammatory cytokines, which characterizes RCC disease. The levels of inflammatory cytokines, including IFN-α, IL-2 and IFN-γ, are significantly decreased in patients with RCC, although the mechanisms are not completely understood. TIPE2 expression in mouse macrophages negatively regulates NF-κB signaling, inhibiting the secretion of certain inflammatory cytokines. TIPE2-knockout and -knockdown macrophages in mice are hypersensitive to TLR stimulation, producing more IL-6 and IL-12 compared with wild-type macrophages (5). Therefore, the upregulation of TIPE2 expression in renal cancer tissue from patients with RCC may be wholly or partially responsible for the reduced levels of inflammatory cytokines.

Clinical evidence has shown that the administration of cytokines leads to regression in certain patients with RCCs. IFNs are glycoproteins that exert antiproliferative effects on tumor cell growth, as well as immunomodulatory and antiviral effects (22). IFN-I induces the unabated activation of peripheral dendritic cells (pDCs), which are recognized as efficient stimulators of B and T lymphocytes and key controllers of immunity and tolerance (23–25). Since the IFN-I family includes 14 IFN-α subtypes and IFN-β, and all subtypes bind to a single receptor, the serum levels of IFN-I may be represented by the mRNA expression levels of MX1, an IFN-I inducible gene (13,26,27). The present study further analyzed the correlation between MX1 and TIPE2 expression in RCC patients and consequently identified a negative correlation between them. This result indicates that there is a close association between the production of IFN-I and TIPE2 expression. Although the production of IFN-I and proinflammatory cytokines requires TLR stimulation, the signaling pathway producing IFN-I downstream of TLR differs from that producing proinflammatory cytokines. IFN regulatory factors (IRFs) are required for the production of IFN-I, while NF-κB is required for the production of proinflammatory cytokines (28,29). Further study is required in the future with regard to whether TIPE2 is able to affect the signaling pathway producing IFN-I.

A question remains concerning the mechanisms involved in the overexpression of TIPE2 mRNA in RCC patients. The etiology of RCC involves environmental and genetic factors. The altered expression of certain immune homeostasis-maintaining genes is ascribed to genetic polymorphisms (4). As for the TIPE2 gene, the upregulation of TIPE2 mRNA expression in renal tumor tissues from RCC patients may also be due to gene polymorphisms. Therefore, investigations into the association between the SNPs of the TIPE2 genes and RCC pathogenesis are eagerly anticipated.

In the present study, the TIPE2 mRNA expression of renal tumor tissues from RCC patients was analyzed prior to treatment with any anti-tumor drugs. The emphasis of our next study will be on investigating the potential effects of anti-tumor drugs and surgical intervention on TIPE2 expression, and assessing the stability of the TIPE2 expression profile over time in individual patients.

In conclusion, the presence of one or several gene amplifications may have prognostic significance (30). The present study indicates that the mRNA expression levels of TIPE2 are increased in renal tumor tissue from RCC patients compared with controls and positively correlated with TNM staging, but negatively correlated with the levels of IFN-I. These findings suggest a significant role for the TIPE2 gene in the pathogenesis of RCC.

## Figures and Tables

**Table I.** Characteristics of the studied subjects.

Characteristic	RCC patients (n=46)	Healthy controls (n=39)
Female, n (%)	20 (43.47)	16 (41.03)
Male, n (%)	26 (56.53)	23 (58.97)
Age (years)	52.4±13.1	49.2±12.8
LDH (U/l)	129±30	115±32
Ca (mmol/l)	2.47±0.68	2.29±0.67
BUN (mmol/l)	6.2±1.3	5.9±1.4
Cr (*μ*mol/l)	81±24	74±19
RBC (10^12^ cells/l)	3.46±1.23	4.42±1.17
WBC (10^9^ cells/l)	5.63±1.99	5.87±2.16
HGB (g/l)	120±25	118±23
PLT (10^9^ cells/l)	201±72	197±90
PT (sec)	12.30±2.40	11.59±2.63
APTT (sec)	26.40±3.30	24.40±3.79
ESR (mm/h)	18±6	14±4
TNM staging, n (%)		
I	12 (26.08)	-
II	10 (21.73)	-
III	18 (39.13)	-
IV	6 (13.04)	-

Values are mean ± standard deviation, unless stated. RCC, renal cell cancer; LDH, lactate dehydrogenase; Ca, calcium; BUN, blood urea nitrogen; Cr, creatinine; RBC, red blood cells; WBC, white blood cells; HGB, hemoglobin; PLT, platelets; PT, prothrombin time; APTT, activated partial thromboplastin time; ESR, erythrocyte sedimentation rate.
